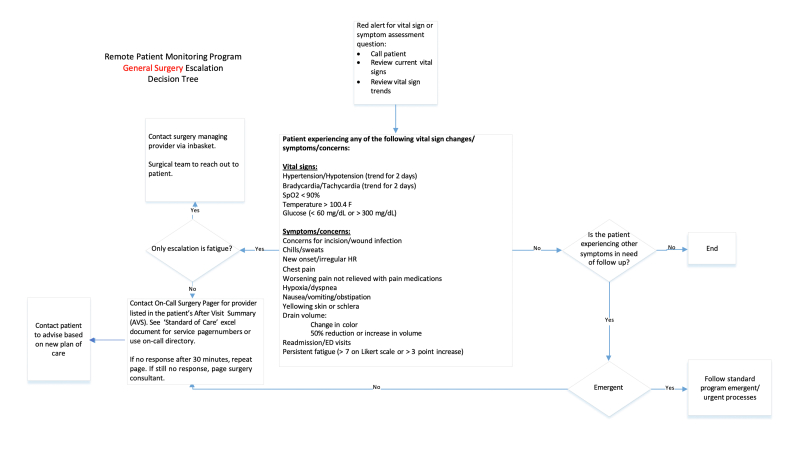# Postsurgical Remote Patient Monitoring Outcomes and Perceptions: A Mixed-Methods Assessment

**DOI:** 10.1016/j.mayocpiqo.2022.09.005

**Published:** 2022-10-21

**Authors:** Aaron Spaulding, Erica Loomis, Emily Brennan, Diane Klein, Karlyn Pierson, Rochelle Willford, M. Susan Hallbeck, Janani Reisenauer

**Affiliations:** a Division of Health Care Delivery Research, Mayo Clinic, Jacksonville, FL; b Robert D. and Patricia E. Kern Center for the Science of Health Care Delivery, Mayo Clinic, Rochester, MN; c Department of Surgery, Mayo Clinic, Rochester, MN; d Center for Digital Health, Mayo Clinic, Rochester, MN; e Department of Surgery Clinical Research Office, Mayo Clinic, Rochester, MN; f Remote Monitoring, Mayo Clinic, Rochester, MN; g Division of Health Care Delivery Research, Mayo Clinic, Rochester, MN

**Keywords:** ASA, American Society of Anesthesiologists, ED, emergency department, ECOG, Eastern Cooperative Oncology Group, LOS, length of stay, RPM, remote patient monitoring, SD, standard deviation

## Abstract

**Objective:**

To determine how postsurgical remote patient monitoring (RPM) influences readmissions and emergency visits within 30 days of discharge after operation and to understand patient and surgeon perspectives on postsurgical RPM.

**Patients and Methods:**

This study was conducted at a US tertiary academic medical center between April 1, 2021, and December 31, 2021. This mixed-methods evaluation included a randomized controlled trial evaluation of RPM after operation and a qualitative assessment of patients’ and surgeons’ perceptions of RPM’s acceptability, feasibility, and effectiveness.

**Results:**

A total of 292 patients participated in the RPM trial, and 147 were assigned to the RPM intervention. Despite a good balance between the groups, results indicated no difference in primary or secondary outcomes between the intervention and control groups. The qualitative component included 11 patients and 9 surgeons. The overarching theme for patients was that the program brought them peace of mind. Other main themes included technological issues and perceived benefits of the RPM platform. The major themes for surgeons included identifying the best patients to receive postsurgical RPM, actionable data collection and use, and improvements in data collection needed.

**Conclusion:**

Although quantitative results indicate no difference between the groups, postsurgical RPM appears well-accepted from the patient’s perspective. However, technological issues could eliminate the benefits. Hospitals seeking to implement similar programs should carefully evaluate which populations to use the program in and seek to collect actionable data.

Over the past decade, a centralized shift has emerged toward implementing mobile technology into the health care system to improve patient care and efficiency. Its use was further propelled by the COVID-19 pandemic, which saw the rapid adoption of telemedicine services.^1^^,^^2^ The impetus for the current research is that needed interventions can be recommended in an earlier, remote fashion that may result in earlier postsurgical discharge, prevent decompensation, and reduce readmissions.

Previous research supports the potential benefits of postsurgical remote patient monitoring (RPM). Siddika et al^3^ reported on 900 patients who underwent colorectal operation over a 9-year period with remote follow-up. They reported substantial financial savings, a 75% reduction in clinic appointments, and a 97% patient satisfaction score. Additionally, a recent meta-analysis conducted by Dawes et al^4^ including 45 remote monitoring studies focused on mobile telemonitoring, reported broad adherence rates, improved quality of life, and decreased readmissions and emergency department (ED) visits. However, they note limitations, including that only 11 studies identified postoperative outcomes, heterogeneity of outcome measures, and weak study designs.

A recent scoping review of digital health solutions has revealed challenges associated with patient adherence, the suitability of digital solutions, and the ability for solutions to be used in different locations.^5^ From the clinical perspective, reduced interaction with patients, the potential for abuse, and a potential increase in care fragmentation have historically slowed the use of telehealth services.^6^ More specifically, clinical concerns surrounding the ability to acquire the required data remotely^7^ and fears of overloaded patient-to-provider messaging have reduced the enthusiasm of the approach for some.^8^

These studies suggest that although there are benefits, challenges relating to our knowledge of the benefits of RPM, usefulness, adherence, and perceptions of benefits with RPM programs also exist. Therefore, the aims of this study were as follows:

• To determine how postsurgical RPM influences readmissions and ED visits within 30 days of discharge after operation.
• To understand patient and surgeon perspectives concerning postsurgical RPM.
• To examine the rates of 30-day complications, including deep vein thrombosis, pulmonary embolism, myocardial infarction, cerebrovascular accident, renal failure, pneumonia, urinary tract infection, surgical site infection, and postoperative length of stay (LOS).

## Patients And Methods

This study was approved by the internal institutional review board and was conducted between April and December 2021. The study occurred in the Department of Surgery at an academic tertiary referral center in the United States. We prospectively enrolled patients who provided informed consent to participate. The inclusion criteria included adults (≥18 years) who were candidates for one of the following inpatient elective operating procedures: abdominal wall reconstruction, gastric bypass, hepatectomy, pancreatectomy, aortic operation, lower extremity bypass, esophagectomy, and colectomy. Elective procedures were chosen so that the patients could be consented in a setting without having received any pain medication or anesthetic in the hospital, which has the potential to impair the ability to consent. Operations performed in a more urgent setting tend to have higher morbidity and mortality rates, which would be a different population to be studied in the future.

Patients were excluded if they had uncontrolled mental illness and/or drug or alcohol abuse; resided in a long-term care facility; were being actively followed for dialysis or transplant services; were pregnant; were being actively treated for cancer; were identified as end-of-life; or had dementia, cognitive impairment, or physical condition that limited their ability to use home remote monitoring equipment independently or interact with the RPM staff.

### Remote Monitoring Intervention

Patients were randomly assigned to the current care or the remote monitoring group at the time of operation, with stratification by age and Eastern Cooperative Oncology Group (ECOG) status.^9^ Patients were followed up for 30 days after discharge, and outcomes were compared between the 2 groups.

The 30-day postdischarge intervention included the provision of a digital tablet and digital blood pressure cuff, thermometer, weight scale, and pulse oximeter. During the 30-day monitoring, patients were asked to collect and report blood pressure, blood oxygen level, temperature, weight, and concerns regarding incision/wound infection, chills, incision/wound infection, new-onset/irregular heart rate, chest pain, worsening pain not relieved with available pain medications, nausea/vomiting/obstipation, jaundice, and scleral icterus drain volume and color changes. If any of these measures fell outside normal parameters or the patient did not send in the measures, they received a nurse call or were escalated to receive appropriate care (Supplemental Figure 1, available online at http://www.mcpiqojournal.org).

The primary outcomes of interest were readmissions and ED visits within 30 days of discharge. Secondarily, we examined rates of 30-day complications, including deep vein thrombosis, pulmonary embolism, myocardial infarction, cerebrovascular accident, renal failure, pneumonia, urinary tract infection, surgical site infection, and postoperative LOS. Complications were defined using the *International Classification of Diseases, Tenth Revision,* diagnosis codes in billing and problem list records.

### Qualitative Evaluation

The qualitative program evaluation included 2 cohorts: patients and surgeons. To be included, patients must have had received remote monitoring after their operation. All surgeons with patients enrolled in the RPM program could participate in this study.

All participants consented, and interviews were conducted to understand perceptions, understanding, acceptability, feasibility, effectiveness, and potential areas for improvement. Patients and physicians were randomly selected and contacted by the study staff to schedule a 30-minute interview via phone or video call. Two staffs of the study team conducted all interviews. Additionally, all patients received a 17-question survey at the end of the 30-day monitoring period. All questions were on a 5-point Likert scale (strongly disagree = 1, strongly agree = 5) concerning their perceptions of the remote monitoring program.

### Statistical Analyses

Continuous variables are presented descriptively as median and range, whereas categorical variables are presented as frequency and percentage. Kruskal-Wallis tests evaluated continuous variables, and Fisher exact tests were used for categorical variables. *P* values of <.05 were considered statistically significant; all tests were 2-sided, and adjustment for multiple testing was not made because of the study’s exploratory nature. All statistical analyses were performed using the R Statistical Software (version 4.0.3; R Foundation for Statistical Computing).

All interviews were audio-recorded, with permission, transcribed verbatim and deidentified for analysis. Three team members analyzed the interview transcripts using directed content analysis. The study team coded the interviews using NVivo qualitative software. The analysis began after 5 interviews were completed and was continued iteratively. Themes were tracked and analyzed using unstructured thematic analyses. Researchers conducted the interviews until thematic saturation was achieved.^10^

## Results

### Quantitative Results

There were 292 patients who participated in the study. The average age was 56.3 years, 45.2% were men, 93.3% identified as White, and 96.5% identified as non-Hispanic or Latino. The average body mass index (calculated as the weight in kilograms divided by the height in meters squared) was 30.6 kg/m^2^; 77.7% had an ECOG score of 0, and only 2.4% of the sample had an American Society of Anesthesiologists (ASA) score of 4 or higher. Of those, 147 were assigned to the RPM group. Table 1 shows the study balance between patient characteristics. The results indicated no difference between the 2 groups’ categories, suggesting adequate balance for comparison.

**Table 1:** Demographic and Clinical Characteristics of Remote Patient Monitoring and Control Patients^a^

Characteristics	No remote patient monitoring (n=145)	Remote patient monitoring (n=147)	Total (N=292)	*P* value^b^
Age at operation (y), median (range)	58.0 (21.0-83.0)	57.0 (22.0-80.0)	57.5 (21.0-83.0)	.66
Male sex, n (%)	63 (43.4)	69 (46.9)	132 (45.2)	.55
Race, n (%)				.28
White	135 (95.7)	129 (90.8)	264 (93.3)	
African, American, or Caribbean Black	4 (2.8)	8 (5.6)	12 (4.2)	
Other	2 (1.4)	5 (3.5)	7 (2.5)	
Ethnicity, n (%)				.34
Non-Hispanic or Latino	134 (95)	138 (97.9)	272 (96.5)	
Hispanic or Latino	7 (5)	3 (2.1)	10 (3.5)	
Operation group, n (%)				.56
Abdominal wall reconstruction	11 (7.6)	6 (4.1)	17 (5.8)	
Bariatrics	5 (3.4)	9 (6.2)	14 (4.8)	
Hepatectomy	17 (11.7)	13 (8.9)	30 (10.3)	
Pancreatectomy	29 (20)	23 (15.8)	52 (17.9)	
Colectomy	47 (32.4)	55 (37.7)	102 (35.1)	
Aortic operation	2 (1.4)	1 (0.7)	3 (1)	
Lower extremity bypass	4 (2.8)	3 (2.1)	7 (2.4)	
Esophagectomy	10 (6.9)	13 (8.9)	23 (7.9)	
Gastrectomy	14 (9.7)	17 (11.6)	31 (10.7)	
Lobectomy	3 (2.1)	2 (1.4)	5 (1.7)	
HIPEC	2 (1.4)	0 (0.0)	2 (0.7)	
Other	1 (0.7)	4 (2.7)	5 (1.7)	
Body mass index (kg/m^2^), median (range)	28.6 (16.1-93.8)	29.1 (18.3-61.1)	28.9 (16.1-93.8)	.47
ECOG score, n (%)				.74
0	114 (79.7)	109 (75.7)	223 (77.7)	
1	21 (14.7)	26 (18.1)	47 (16.4)	
2	6 (4.2)	8 (5.6)	14 (4.9)	
3	2 (1.4)	1 (0.7)	3 (1.0)	
ASA score, n (%)				
1	0 (0.0)	1 (0.7)	1 (0.3)	
2	58 (40.3)	59 (40.7)	117 (40.5)	
3	82 (56.9)	82 (56.6)	164 (56.7)	
4	4 (2.8)	3 (2.1)	7 (2.4)	
5	0 (0.0)	0 (0.0)	0 (0.0)	
Diabetes, n (%)	24 (16.7)	28 (19.2)	52 (17.9)	.58
Hypertension, n (%)	59 (41.0)	67 (45.9)	126 (43.4)	.40
Charlson score (without diabetes), median (range)	2.0 (0.0-6.0)	2.0 (0.0-5.0)	2.0 (0.0-6.0)	.14
Wound classification, n (%)				.42
Type I clean	11 (7.6)	6 (4.1)	17 (5.9)	
Type II clean-contaminated	110 (76.4)	112 (76.7)	222 (76.6)	
Type III contaminated	19 (13.2)	20 (13.7)	39 (13.4)	
Type IV dirty or infected	4 (2.8)	8 (5.5)	12 (4.1)	
No. of operations in admission, n (%)				.75
1	136 (94.4)	135 (92.5)	271 (93.4)	
2	5 (3.5)	7 (4.8)	12 (4.1)	
3	0 (0.0)	2 (1.4)	2 (0.7)	
4	2 (1.4)	1 (0.7)	3 (1.0)	
5	1 (0.7)	1 (0.7)	2 (0.7)	
Postoperative ICU, n (%)	14 (9.7)	14 (9.5)	28 (9.6)	.97
Postoperative ICU hours, median (range)	32.0 (15.0-628.0)	43.5 (18.0-141.0)	41.0 (15.0-628.0)	.41

a ASA, American Society of Anesthesiologists; ECOG, Eastern Cooperative Oncology Group; HIPEC, Hyperthermic Intraperitoneal Chemotherapy; ICU, intensive care unit.

b *P* values result from Kruskal-Wallis tests for continuous variables and from chi-square tests for categorical variables.

Table 2 demonstrates no differences in the readmission rate (RPM: 19.7% vs no RPM: 20.7%, *P*=.84) or ED visits (RPM: 6.8% vs no RPM: 7.6%, *P*=.80). Additionally, there were no differences in the secondary outcomes (*P*>.05). The patients in the RPM group exhibited a mean of 86% adherence with daily vitals logging and 78% adherence with daily question logging.

**Table 2:** 30-Day Primary and Secondary Remote Patient Monitoring Outcomes

Outcomes	No remote patient monitoring (n=145)	Remote patient monitoring (n=147)	Total (N=292)	*P* value^a^
Any readmission in 30 d, n (%)	30 (20.7)	29 (19.7)	59 (20.2)	.84
24-hour readmission in 30 d, n (%)	16 (11.0)	12 (8.2)	28 (9.6)	.40
Emergency department visit in 30 d, n (%)	11 (7.6)	10 (6.8)	21 (7.2)	.80
Postoperative length of stay (h), median (range)	99.8 (2.9-2009.7)	95.9 (5.3-668.2)	96.9 (2.9-2009.7)	.59
Acute deep vein thrombosis in 30 d, n (%)	1 (0.7)	0 (0.0)	1 (0.3)	.50
Acute pulmonary embolism in 30 d, n (%)	0 (0.0)	0 (0.0)	0 (0.0)	
Myocardial infarction in 30 d, n (%)	0 (0.0)	0 (0.0)	0 (0.0)	
Cerebrovascular accident in 30 d, n (%)	0 (0.0)	1 (0.7)	1 (0.3)	.99
Renal failure in 30 d, n (%)	1 (0.7)	0 (0.0)	1 (0.3)	.31
Pneumonia in 30 d, n (%)	1 (0.7)	0 (0.0)	1 (0.3)	.50
Urinary tract infection in 30 d, n (%)	0 (0.0)	0 (0.0)	0 (0.0)	
Surgical site infection in 30 d, n (%)	3 (2.1)	5 (3.4)	8 (2.7)	.72

a *P* values result from Kruskal-Wallis tests for continuous variables and from chi-square tests for categorical variables.

### Survey Results

A total of 52 patients responded to the postsurgical remote monitoring survey (35% response rate, Table 3). The survey revealed that patients agreed (average, 4.6/5) with each question. When considering overall satisfaction with the program, patients scored 4.7/5 (standard deviation [SD], 0.7) and 4.8/5 (SD, 0.7) when asked if they would recommend it to others. Questions with the lowest scores included whether the education materials provided were useful (4.3/5; SD, 0.7), the equipment helped in their care at home (4.4/5; SD, 0.8), and whether the program helped them feel comfortable managing their health at home (4.4/5; SD, 1.0).

**Table 3:** Postsurgical Remote Patient Monitoring Satisfaction Survey

Survey Questions	Overall (N=52)	
	Mean (SD)	Range
The Remote Patient Monitoring Program helped me feel comfortable managing my health at home	4.4 (1.0)	1.0-5.0
The team explained how to use the equipment	4.5 (0.8)	1.0-5.0
The medical equipment was easy to use	4.6 (1.0)	1.0-5.0
The equipment helped in my care at home	4.4 (0.8)	1.0-5.0
I felt comfortable interacting with the team by phone or tablet	4.8 (0.6)	1.0-5.0
The team explained things to me in a way that was easy to understand	4.6 (0.7)	1.0-5.0
The team listened to my concerns	4.6 (0.8)	1.0-5.0
The team kept me informed about my care plan	4.6 (0.8)	1.0-5.0
I was able to reach a member of the team right away for any questions or concerns	4.5 (0.9)	2.0-5.0
The team promptly responded to my needs	4.5 (0.7)	3.0-5.0
The team explained when I should seek medical attention	4.5 (0.8)	3.0-5.0
The educational materials provided by the team were useful	4.3 (0.7)	3.0-5.0
I felt ready to leave the Remote Patient Monitoring program	4.7 (0.7)	1.0-5.0
The team treated me with courtesy and respect	4.9 (0.6)	1.0-5.0
The staff worked well together to care for me	4.8 (0.7)	1.0-5.0
I would recommend the Remote Patient Monitoring Program to others with a similar health condition(s)	4.8 (0.7)	1.0-5.0
Overall, I am satisfied with the Remote Patient Monitoring Program	4.7 (0.7)	1.0-5.0

### Qualitative Results

#### Patient Interviews

There were 11 patients interviewed, and their average age was 57 years (average age of all trial participants was 56 years). They had a variety of surgical procedures performed in different specialties (Table 4). The overarching theme was that the program brought them peace of mind, and other major themes included technological issues and the perceived benefits of the RPM.

**Table 4:** Patient Demographic Characteristics

Age range	Surgical procedure group
50-60	Colorectal operation
50-60	Bariatrics
60-70	Hepatectomy
80-90	Colorectal operation
30-40	Gastrectomy
60-70	Pancreatectomy
50-60	Colorectal operation
40-50	Colorectal operation
60-70	Bariatrics
50-60	Colorectal operation
60-70	Pancreatectomy

##### Peace of Mind

When queried about the program, most patients indicated the main benefit was the peace of mind. For example, 1 patient suggested *“… just knowing that my O*_*2*_ *level was good and my heart rate was good, it gave me peace of mind”* (P1). Some of the peace of mind was directly attributable to knowing that the data collected were being reviewed: *“There was one day I had eaten a popsicle and go for my vitals, not thinking about taking my temperature. So, my temperature was really low, and they actually called to check on me”* (P4). Finally, 1 patient indicated *“It was just nice to see that you’re improving and that they’re monitoring you, especially right after surgery”* (P11).

##### Technology Issues

About half of the patients mentioned that they needed changes to the equipment or having connection issues. For example, 1 patient indicated that they had difficulty using the thermometer and reading it: *“It was a pain to use… I always felt like if you put it in one place in your mouth, you didn’t get the same consistency– and it was hard to read”* (P9). Another patient mentioned trouble uploading the data after they had recorded all the data into the program: *“….on that pad, then you’d select all, and then it checkmarks all your vitals – but then, when you go to send it, a lot of times, it won’t go”* (P3). Another suggested it wasn’t the equipment, but the connection: *“It wasn’t the equipment not working. It was the connection to the tablet to send it”* (P10)*.*

##### Perceived Benefits of the RPM Platform

All patients believed that the program was effective: *“the main benefit was just to monitor my vitals to make sure that I wasn’t getting sick…I didn’t have a fever, or my blood pressure wasn’t getting goofy”* (P6). They suggested that they used this information not to overexert themselves: *“…so then that way, I wasn’t overdoing myself as far as trying to do things around the house and that kind of stuff”* (P6). Finally, 1 patient credited the program for helping her identify the need for new blood pressure medications: *“Well, I had some issues with lower blood pressure and some heart rate issues, and I don’t think we would have ever discovered those had we not done that”* (P9).

#### Surgeon Interviews

Overall, the 9 surgeons interviewed had been on consulting staff at the institution for an average of 10.7 years (Table 5). The following major themes were identified from the interviews: identifying the best patients to receive postsurgical RPM (yield), actionable data collection and use, and improvements in data collection needed.

**Table 5:** Surgeon Specialty

Specialty
Colorectal operation
Thoracic operation
Hepatobiliary
Vascular and endovascular operation
Thoracic operation
Colorectal operation
Hepatobiliary and pancreatic operation
General operation
General operation

##### Identifying the Best Patients to Receive Postsurgical RPM

When asked which patients would be best to receive RPM, physicians often responded in the context of risk stratification. Most followed by indicating a more complex case or more complex patient would be better to track over time as they were at higher risk of complication or readmission. *“I think the lowest hanging fruit would be in the patient population at highest risk for readmission…the walking well that have a straightforward operation, those patients, you’ll never see them again”* (S9). In contrast, less-complicated procedures or less-complicated patients might not need monitoring: “*… if you have a procedure that has very low risk of complication, very low risk of readmission, probably the benefit just is not there, so the cost may not be appropriate”* (S2).

##### Actionable Data

There were multiple mentions of ensuring actual treatment options and a need to measure actionable things. One clinician stated “*…I mean the devil is in the details in terms of what does that really mean, what’s the device, what parameters are you measuring, how useful is it, how long do you need to measure”* (S5). Similarly, another clinician elaborated that the measures would have to provide an opportunity to do something different from the current standards of care: “*I would say it would be patients who have a potential set of complications that can be recognized with sort of standard vitals and standard questions…”* (S4).

##### Improve Types of Data Collected

Surgeon responses indicated opportunities to improve the types of data collected. *“I mean an option would be to have patients send in photos of like their wounds as sort of a routine thing… an objective sort of measure”* (S4). Further, other surgeons suggested aspects such as glucose monitoring and mobility: *“… things like glycemic control because that, obviously, impacts wounds and healing. … Mobility – like if they have a Fitbit or something to see what kind of – how many steps they’re doing. Are they getting up and around?”* (S6).

## Discussion

This study found no differences between outcomes of interest in the RPM and no RPM groups.

Previous studies that have employed techniques for early checkpoints and communication with patients have reported reduced readmission risks.^11^ In particular, a remote surveillance program employed in the United Kingdom in 900 patients decreased costs by 63%, with high patient satisfaction (97%).^3^ Our study differs in that it was conducted on patients who underwent an operation in an outpatient setting in a prospective randomized trial. Further, our current remote monitoring equipment, which has been applied to patients who underwent cardiovascular operation during their inpatient stay, has found ease, practical use, and positive implications in discharge planning and LOS.^12^ As a result, although our study differs regarding outcomes, it provides incremental evidence concerning the use of postsurgical RPM.

A recent meta-analysis analyzed 45 studies in which mobile telemonitoring was performed in patients postsurgery.^4^ Although there was no difference in the number of outpatient visits, there was a significant reduction in hospital (odds ratio: 0.47, 95% confidence interval: 0.29-0.79) and ED visits (odds ratio: 0.42, 95% confidence interval: 0.23-0.79) in the pooled analysis; however, there were no differences in complications. Of note, only a few individual studies have found differences in outcomes between groups, including patients who underwent transplant and total knee and hip arthroplasty. The findings from this meta-analysis prompt questions regarding the current study’s results. Physician interviews provide insight into our nonsignificant findings. The overarching theme focused on finding patients who would benefit most from the program and ensuring that the data were actionable. To this point, there is a likely association with the risk of these events occurring in our patient population. These assertions are supported by the fact that we only included patients receiving elective operation. Further, when reviewing the characteristics of the populations, we see lower ECOG, ASA, and Charlson scores, as well as mostly type II wound classifications. This could point to a limited risk of ED visits, readmissions, and any of the secondary measures assessed.

The current study also provides beneficial insights into patient perspectives. The patients described the program as bringing them peace of mind, despite reporting technical issues. This is consistent with previous findings indicating larger gains in quality of life outcomes in patients exposed to telehealth over those receiving standard care.^4^ Patients are often left to identify potential postsurgical issues with limited understanding of symptoms based on instructions they receive before discharge, after an operation.^13^, ^14^, ^15^ This less-than-ideal situation places patients at the risk of misidentifying potential postsurgical complications and concerns.^16^ As a result, patients often seek care that they do not need or wait until minor issues develop into more substantial complications. Second, postsurgical RPM may help improve patient perspectives regarding their care. After medical or surgical interventions, patient satisfaction measures provide important insight into how patients perceive their care and are often used to rate hospitals and providers.^17^^,^^18^ Further, value-based assessments that utilize patient experience domains have been used to reward organizations for increased patient satisfaction.^19^ As a result, both a patient and organization desire to improve patient perceptions of the care they receive. However, patient reports of technology issues are worrisome for both areas. Specifically, poor technological execution may degrade patient confidence, perceived usefulness, and ease of use.^20^^,^^21^ These perceptions may erode the potential benefits associated with increased care coordination and improved patient satisfaction. Ultimately, patient reports of peace of mind suggest that a previously unmet need was met through RPM; however, technological issues may considerably dampen that enthusiasm.

The results of the study provide insight for future RPM implementation. This approach appears to be accepted from the patient’s perspective, which was likely a considerable hurdle historically. However, those who would implement RPM technology should ensure that the technology functions appropriately and does not reduce patient confidence in the technologies or structures provided. Additionally, creating robust, more straightforward plug-and-play devices is important for gaining the trust and involvement of patients. These aspects could promote improved self-efficacy, patient-provider relationships, and even postsurgical adherence to discharge instructions.^22^^,^^23^ Next are opportunities to risk-stratify patients to identify those with risk of adverse events. Although the current study does not identify specific metrics, risk calculators could be used for stratification, including those focused on readmissions,^24^ or complications.^25^^,^^26^ Additionally, procedure-specific measures associated with wound complications, the complexity of the procedure,^27^ or patient variables such as cognitive ability, mental health, social support, or health literacy could be used to help identify RPM need.^28^ Future studies should evaluate these opportunities as the current study results suggest some risk categorization will likely improve the cost-benefit of using RPM technologies after operation.

This study has some limitations. This study was conducted at a single institution, and as a result, the results are linked to the processes and protocols that existed during the study. Further, how RPM is implemented and which patients receive the services likely influence the subsequent outcomes. Additionally, responses could differ among patients who agreed or declined to participate. However, differences between those populations will need to be evaluated in future studies because the declination of study consent restricted our ability to ascertain patient reasons for refusal. Finally, additional methods of inquiry and additional patient and provider interviews could reveal further insight. Nevertheless, we have followed appropriate methodological guidelines and sought to understand better the perspectives of patients and physicians regarding postsurgical RPM. Therefore, we recommend future studies build on the current research and seek further knowledge regarding improvements and perceptions of similar programs.

## Conclusion

This study sought to assess changes in readmissions and ED visits within 30 days of discharge after operation and to understand patient and surgeon perspectives on postsurgical RPM. Although the results indicated no difference in readmission or ED visits, postsurgical RPM appears well-accepted from the patient’s perspective. Nevertheless, technological issues could eliminate the benefits. Hospitals seeking to implement similar programs should carefully evaluate which populations to use the program in and seek to collect actionable data.

## Potential Competing Interests

The authors report no competing interests.

## Figures and Tables

**Supplemental Fig. S1:**